# Intrahepatic bile duct stone removal using a tapered-tip sheath system

**DOI:** 10.1055/a-2601-0175

**Published:** 2025-06-18

**Authors:** Fumioki Toyoda, Yuya Muramoto, Tomoaki Matsumori, Masataka Yokode, Hiroshi Seno

**Affiliations:** 1 Department of Gastroenterology and Hepatology, Kyoto University Graduate School of Medicine, Kyoto, Japan

A 64-year-old man with hilar biliary strictures following liver transplantation was admitted for a scheduled biliary stent exchange. During initial endoscopic retrograde cholangiopancreatography (ERCP), a filling defect on the peripheral side of the B6 branch stricture was observed ( Fig. 1 **a, b** ), indicating intrahepatic bile duct (IHBD) stones. The stricture was dilated using a 6-mm dilation balloon catheter (REN; Kaneka Co., Inc.) ( Fig. 1 **c** ), and 5 Fr endoscopic nasobiliary drainage tubes (SilkyPass J type, Boston Scientific Co.) were inserted. Stones were removed during the second ERCP session. A basket catheter (Medi-Globe 8-Wire Nitinol Basket; Medico’s Hirata Inc) was inserted over the guidewire (EndoSelector, Boston Scientific Co.) ( Fig. 2 **a** ); however, not all of the stones were removed ( Fig. 2 **b** ). A three-layered mechanical lithotripter (Xemex Crusher Catheter LBMT320; Zeon Medical) was used for the larger stones but failed to pass through the stricture ( Fig. 2 **c** ). Therefore, a tapered tip sheath system (EndoSheather, Piolax), composed of a tapered inner catheter and an outer sheath ( Fig. 3 **a, b** ), was advanced over the guidewire and positioned at the periphery of the remaining filling defects. The inner sheath and guidewire were withdrawn ( Fig. 4 **a** ), and the three-layered inner basket of the lithotripter was inserted through the outer sheath ( Fig. 4 **b** ). The remaining stones were captured ( Fig. 4 **c** ), achieving complete stone removal ( Fig. 4 **d** , Video 1 ).

**Fig. 1:**
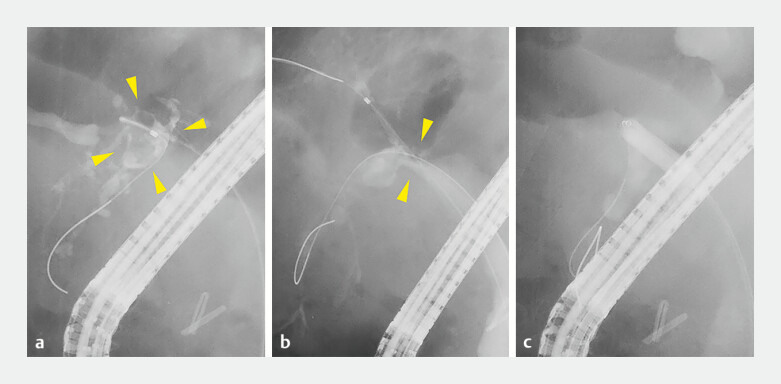
The images of the initial endoscopic retrograde cholangiopancreatography (ERCP) procedure. A cholangiography image showing a filling defect ( **a** yellow arrowheads) on the peripheral side of the B6 branch stricture ( **b** yellow arrowheads). **c** The B6 branch stricture was dilated using a 6-mm dilation balloon catheter (REN; Kaneka Co., Inc.).

**Fig. 2:**
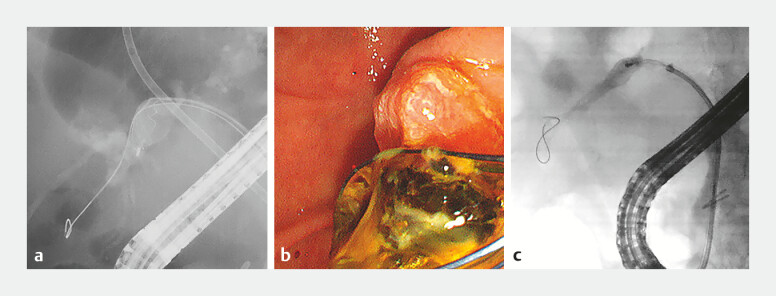
The images of the second ERCP procedure. **a** A basket catheter (Medi-Globe 8-Wire Nitinol Basket; Medico Hirata Inc.) was inserted into the B6 branch. **b** Some stones were successfully removed using a basket catheter. **c** A three-layered mechanical lithotripsy basket (Xemex Crusher Catheter LBMT320; Zeon Medical) failed to pass through the stricture or angulation of the B6 branch.

**Fig. 3:**
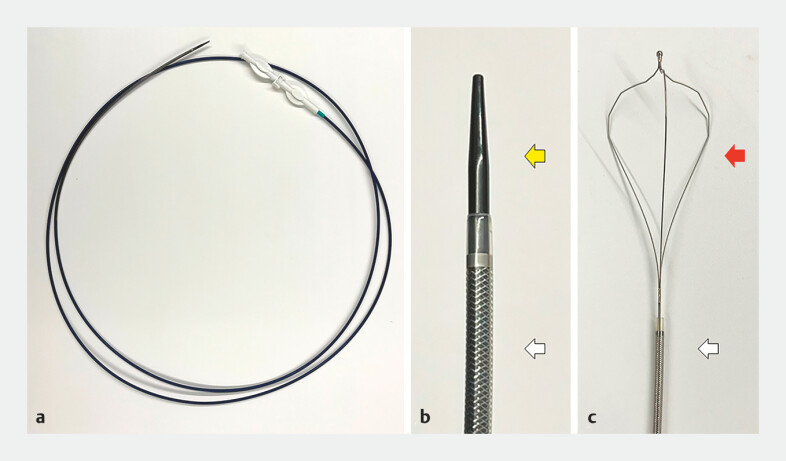
Images of the tapered-tip sheath system. **a** Overview of the tapered-tip sheath system (EndoSheather, Piolax). **b** The tapered-tip inner catheter tip (a yellow arrow) and an outer sheath (a white arrow). **c** Tip of the inner basket of the lithotripter (Xemex Crusher Catheter LBMT320; Zeon Medical) (a red arrow) and an outer sheath (a white arrow).

**Fig. 4:**
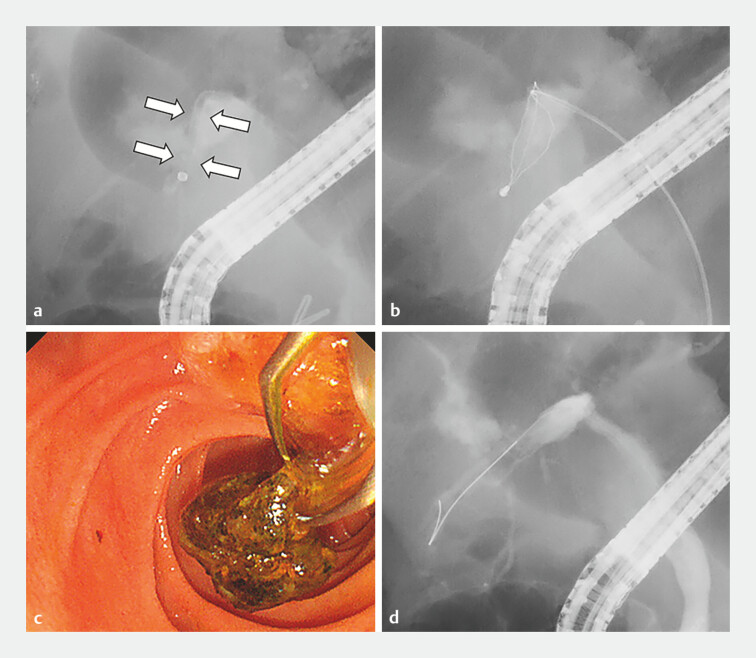
Stone removal using the tapered-tip sheath system. **a** The tapered-tip sheath system was inserted over the guidewire to the peripheral side of the remaining filling defects. Thereafter, the inner sheath and the guidewire were withdrawn (white arrows). **b** The three-layered inner basket was inserted through the outer sheath. **c** The basket successfully recovered the remaining stones. **d** Complete removal of the B6 stones was achieved.

This video shows the efficient procedure for intrahepatic bile duct stone removal using a tapered-tip sheath system.Video 1

Biliary strictures are common complications of liver transplantation leading to IHBD stone formation distal to the stricture 1 . Endoscopic transpapillary stone removal can be challenging because of bile duct angulation and strictures 2 . The tapered-tip sheath system, originally developed for bile duct biopsy 3 , enabled the deployment of the basket along the same axis as the bile duct, facilitating efficient stone retrieval and improving the efficiency of IHBD stone removal.

Endoscopy_UCTN_Code_TTT_1AR_2AH

## Notes

Endoscopy E-Videos https://eref.thieme.de/e-videos
*E-Videos* is an open access online section of the journal *Endoscopy* , reporting on interesting cases and new techniques in gastroenterological endoscopy. All papers include a high-quality video and are published with a Creative Commons CC-BY license. Endoscopy E-Videos qualify for HINARI discounts and waivers and eligibility is automatically checked during the submission process. We grant 100% waivers to articles whose corresponding authors are based in Group A countries and 50% waivers to those who are based in Group B countries as classified by Research4Life (see: https://www.research4life.org/access/eligibility/ ). This section has its own submission website at https://mc.manuscriptcentral.com/e-videos .
